# Systems Biology Approach to Identify Novel Genomic Determinants for Pancreatic Cancer Pathogenesis

**DOI:** 10.1038/s41598-018-36328-w

**Published:** 2019-01-15

**Authors:** Indu Khatri, Koelina Ganguly, Sunandini Sharma, Joseph Carmicheal, Sukhwinder Kaur, Surinder K. Batra, Manoj K. Bhasin

**Affiliations:** 1 BIDMC Genomics, Proteomics, Bioinformatics and Systems Biology Center, Beth Israel Deaconess Medical Center, Boston, MA USA; 2 Department of Biochemistry and Molecular Biology, University of Nebraska Medical Center, Omaha, Nebraska USA

## Abstract

Pancreatic ductal adenocarcinoma (PDAC) is a lethal malignancy with a 5-year survival rate of <8%. Its dismal prognosis stems from inefficient therapeutic modalities owing to the lack of understanding about pancreatic cancer pathogenesis. Considering the molecular complexity and heterogeneity of PDAC, identification of novel molecular contributors involved in PDAC onset and progression using global “omics” analysis will pave the way to improved strategies for disease prevention and therapeutic targeting. Meta-analysis of multiple miRNA microarray datasets containing healthy controls (HC), chronic pancreatitis (CP) and PDAC cases, identified 13 miRNAs involved in the progression of PDAC. These miRNAs showed dysregulation in both tissue as well as blood samples, along with progressive decrease in expression from HC to CP to PDAC. Gene-miRNA interaction analysis further elucidated 5 miRNAs (29a/b, 27a, 130b and 148a) that are significantly downregulated in conjunction with concomitant upregulation of their target genes throughout PDAC progression. Among these, miRNA-29a/b targeted genes were found to be most significantly altered in comparative profiling of HC, CP and PDAC, indicating its involvement in malignant evolution. Further, pathway analysis suggested direct involvement of miRNA-29a/b in downregulating the key pathways associated with PDAC development and metastasis including focal adhesion signaling and extracellular matrix organization. Our systems biology data analysis, in combination with real-time PCR validation indicates direct functional involvement of miRNA-29a in PDAC progression and is a potential prognostic marker and therapeutic candidate for patients with progressive disease.

## Introduction

Pancreatic ductal adenocarcinoma (PDAC) is one of the most lethal solid malignancies with exceptionally low survival and an exceedingly high mortality rate^1^. Its occult nature and the lack of non-invasive sensitive biomarkers result in diagnosis often after the tumor has advanced locally to the point of being nonresectable or metastasized to distant sites^2^. Along with early and expeditious detection of PDAC, proper prognostic stratification of patients predicated on the basis of non-invasive serum markers will also facilitate better usage of novel targets for chemo-preventive strategies. This is a critical step for improving the clinical outcome of PDAC patients Serum-based markers (i.e. CA125 and secretory mucins like MUC5AC) and tissue-based proteins (i.e. macrophage inhibitory cytokine-1, K-ras, mesothelin, PSCA, mucins, SMAD4 and p53 mutations) have been receiving attention as potential PDAC diagnostic and prognostic biomarkers, and many are currently undergoing validation for early detection and prediction of PDAC progression^3^. Elucidating the role of significantly altered molecules in the onset and progression of this disease is essential for understanding the disease specific etiology.

miRNAs, a group of small non-coding RNAs, have emerged recently as potential molecular contributors in carcinogenesis including initiation, progression and metastasis^4^. Cancer-related miRNAs, apart from regulating the expression of known protein-coding oncogenes and tumor suppressors, behave as oncogenes themselves (referred to as oncomirs) as well as acting as tumor suppressors^5^. miRNAs have previously been reported to exhibit exclusively distinct expression profiles in PDAC, chronic pancreatitis (CP) and normal pancreas with each profile associated with distinctive pathology and clinical status^6^. For example, miRNA-21 levels in serum were reported to be significantly associated with overall pancreatic cancer survival^7^.

In order to have a clear insight into the contribution of a circulating molecular signature to determine pancreatic cancer prognosis, analyses should be focused on molecules that show significant distinction in their expression across the progressive spectrum of disease states. Multidimensional analysis of omics data related to miRNAs have assisted in identifying the key therapeutic and prognostic targets for multiple cancers^8^. Supplementary to this, the combination analysis of miRNA and their target genes that are concordantly dysregulated with disease progression, more significantly reflect the true pathophysiology of the disease, as compared to gene or miRNA analysis alone. Understanding the etiology of the differentially expressed miRNAs and their downstream mRNA targets using a systems biology approach will not only lead to a better diagnostic outcome with fewer false positives, but also will enhance the efficiency and possibilities of designing targeted drugs^9^.

In the present study, we explored the combined potency of gene and miRNA datasets collected at different pancreas statuses (Healthy controls(HC), CP, PDAC and metastatic PDAC (MPDAC)), to identify novel prognostic markers associated with the onset and progression of PDAC. To identify a global PDAC signature, we placed emphasis on genes and miRNA that are consistently associated with PDAC in all the data sets, both at the tissue as well as the blood/serum level. Further functional and survival analysis of the identified miRNA-mRNA interacting pairs, identified an association with pathways linked to cancer progression and survival. From our analysis, miRNA-29a/b emerged as the most efficacious prognostic marker with a decreasing enrichment score from CP to PDAC to MPDAC. Thus, indicating its role as a possible tumor suppressor responsible for downregulating genes involved in PDAC pathogenesis. Lastly, quantitative Real-Time PCR analysis from serum samples obtained from healthy individuals, those diagnosed with chronic pancreatitis, early and late stage PC, clearly demonstrated a decreasing trend in the abundance of miRNA-29a/b, thereby corroborating our in-silico analysis.

## Results

### Analytical approach to identify key miRNAs associated with progression to PDAC

We performed an integrative meta-analysis on miRNA and mRNA data from CP and PDAC cases to identify the miRNAs that are putatively associated with pathophysiology of CP to PDAC. In order to identify the miRNAs originating from the pancreas and circulating in blood, we included miRNA studies from both tissue as well as the blood in our meta-analysis. Differentially expressed (DE) miRNAs were identified in a status specific manner (CP, PDAC, MPDAC), along with their associated target mRNAs. The common DE genes obtained across multiple PDAC datasets capitulate heterogeneity across patients and robustly screen disease related genes. The miRNA meta-analysis included the 186 samples from HC, 102 from CP subjects and 226 from PDAC subjects (Table 1). The mRNA dataset has 9 transcriptomic profiles from each patient group: HC, CP, PDAC and MPDAC (Table 1). Supplementary Fig. S1 shows a schematic of the overall methodology used to identify dysregulated miRNAs across the pancreatic disease spectrum.

**Table 1:** List of pancreatic ductal adenocarcinoma *omics* datasets used in this study.

	Dataset	Source	Samples				References	
			HC	CP	PDAC	MPDAC	Publications	PMID
miRNA	GSE24279 (Dataset I)	Tissue	22	27	136	0	Bauer, A. S. *et al*.^77^	22511932
	GSE31568 (Dataset II)	Blood	70	38	45	0	Keller, A. *et al*.^78^	21892151
	GSE61741 (Dataset III)	Blood	94	37	45	0	Keller, A. *et al*.^79^	25465851
mRNA	E-EMBL-6 (Dataset IV)	Tissue	9	9	9	9	Abdollahi, A. *et al*.^80^	17652168

### miRNA meta-signature in progressive pancreatic cancer statuses

Supervised and unsupervised analyses were performed on normalized miRNA datasets from plasma as well as tissue studies. Principal Component Analysis (PCA) results showed a good separation of PDAC and normal samples along the first three principal components (PC) in all datasets. PC1 accounted for 10.64–15.83% of the variance and depicted a significant separation of PDAC and HC. Interestingly, CP samples depicted heterogeneity and overlapped with both normal and PDAC clusters (Supplementary Fig. S2).

**Figure 1:**
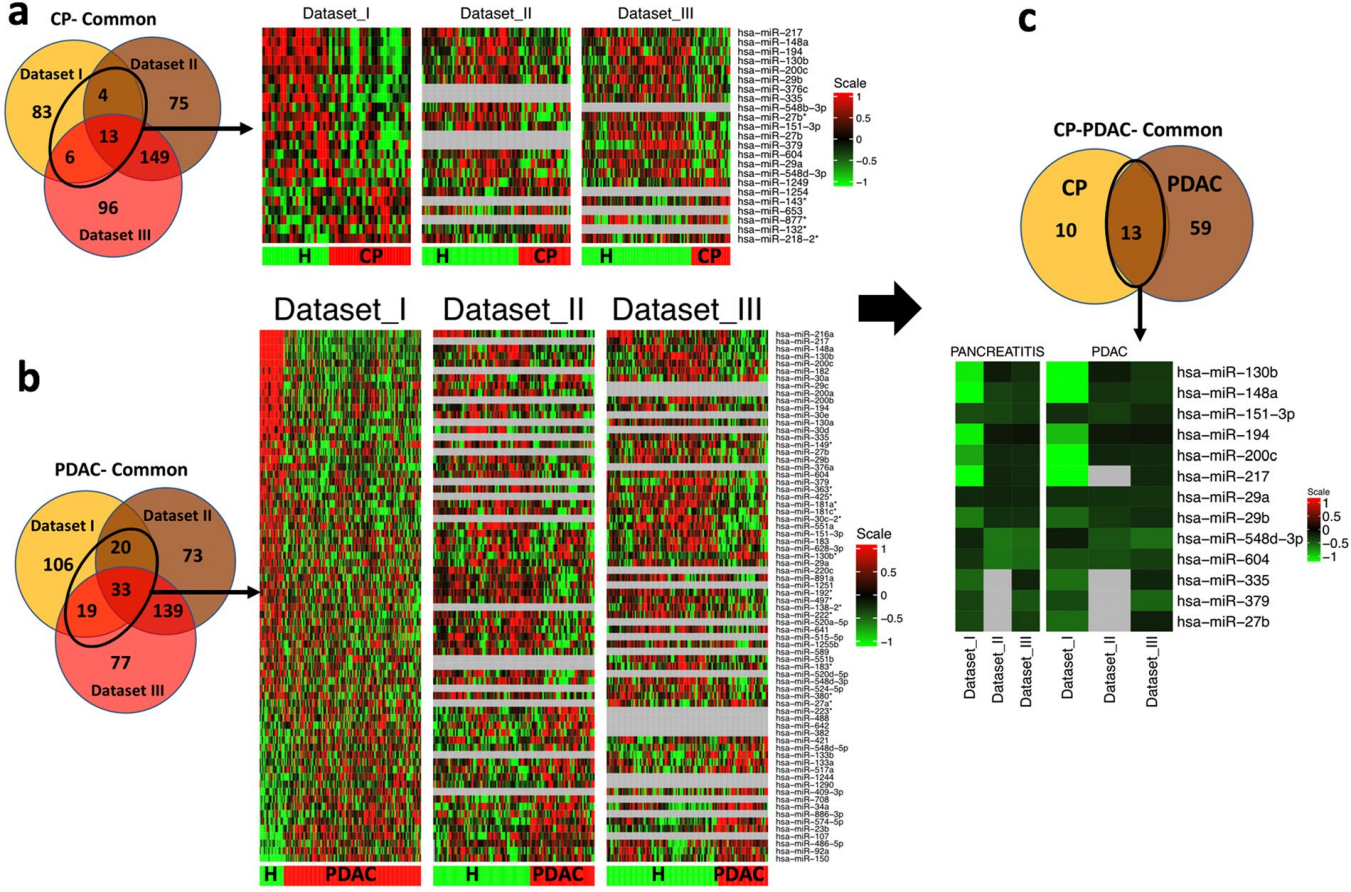
Identification of DE miRNAs commonly dysregulated in CP and PDAC subjects in tissue and blood datasets. (**a**) Venn diagram of the miRNA differentially expressed in HC vs CP conditions and common across Dataset I, II and III; and a heatmap of the miRNA differentially expressed across Dataset I and Dataset II or III where ‘HC’ represents healthy controls and ‘CP’ represent subjects with chronic pancreatitis. (**b**) Venn diagram of the miRNA differentially expressed in HC vs PDAC condition and common across Dataset I, II and III; and the heat map of the miRNA differentially expressed between Dataset I and Dataset II or III where ‘HC’ represents healthy controls and ‘PDAC’ represent subjects with pancreatic ductal adenocarcinoma. (**c**) Venn diagram of the miRNA common in between HC vs CP and HC vs PDAC condition within Dataset I and Dataset II or III.

The miRNA meta-analysis identified 23 consistently and significantly DE miRNAs; 7 upregulated (miRNA-218-2*, miRNA-1249, miRNA-1254, miRNA-653, miRNA-132*, miRNA-143*, miRNA-877*) 16 downregulated (miRNA-27b*, miRNA-548d-3p, miRNA-604, miRNA-148a, miRNA-151-3p, miRNA-29b, miRNA-130b, miRNA-200c, miRNA-217, miRNA-29a, miRNA-194, miRNA-548b-3p, miRNA-376c, miRNA-335, miRNA-379, miRNA-27b) in CP (tissue and plasma) compared to normal, P value ≤ 0.05 (miRNA CP meta-signature). A star (asterisk) is appended to the miRNA name to designate less abundant product of mature miRNA (star strand) that is opposite of guide strand producing predominant miRNA product^10^. A Venn diagram and heatmap of the top DE miRNAs associated with CP is shown in Fig. 1a. Among these commonly altered miRNAs, miRNA-29 loss is correlated with a significant increase in extracellular matrix (ECM) deposition which is a major component in PDAC stroma^11^, miRNA-200c is reported as an independent prognostic factor in pancreatic cancer^12^ and miRNA-143 plays a central role in the invasion and metastasis of pancreatic cancer and is also a potential target for pancreatic cancer therapy^13^.

Further meta-analysis of PDAC vs normal miRNA profiles identified 72 consistently and significantly altered miRNAs (22 upregulated and 50 downregulated), P value ≤ 0.05. These miRNAs depicted consistent dysregulation between blood/plasma and tissues, therefore might be shed by PDAC tissue into the blood Fig. 1b (i.e. PDAC meta-signature). The heatmap clearly depicts the uniform upregulation or downregulation of different miRNAs across-datasets. Thirteen miRNAs were found to be consistently dysregulated both during CP and PDAC conditions in at least 2 out of 3 datasets (Fig. 1c). Interestingly, these common miRNAs were downregulated in tissue and blood samples (dataset 1 versus II and III) as depicted in the heatmap (Fig. 1c). miRNA-130b, 148a, 151-3p, 194, 200c, 217, 29a, 29b, 548d-3p, 604, 335, 379, 27b were all found to be downregulated in CP and PDAC groups as compared to HC, P value < 0.05 (Table 2). Notably, none of the upregulated miRNAs were common in both the groups.

**Table 2:** Log Fold Change and P-values of DE miRNA common in HC vs CP and HC vs PDAC conditions in Dataset I and Dataset II/Dataset III.

	Dataset I				Dataset II				Dataset III			
	CP		PDAC		CP		PDAC		CP		PDAC	
	logFC	P.Value	logFC	P.Value	logFC	P.Value	logFC	P.Value	logFC	P.Value	logFC	P.Value
hsa-miR-130b	−0.93	0.00	−1.66	0.00	−0.14	0.04	−0.14	0.03	−0.24	0.00	−0.27	0.00
hsa-miR-148a	−1.06	0.00	−2.09	0.00	−0.32	0.00	−0.25	0.00	−0.28	0.00	−0.28	0.00
hsa-miR-151-3p	−0.36	0.00	−0.23	0.00	−0.33	0.00	−0.30	0.00	−0.28	0.00	−0.20	0.01
hsa-miR-194	−0.95	0.00	−0.77	0.00	−0.12	0.01	−0.11	0.01	−0.10	0.02	−0.10	0.01
hsa-miR-200c	−0.70	0.00	−1.09	0.00	−0.20	0.04	−0.19	0.04	−0.21	0.03	−0.20	0.02
hsa-miR-217	−1.19	0.00	−2.42	0.00	−0.19	0.04	NA	NA	−0.21	0.01	−0.20	0.00
hsa-miR-29A	−0.19	0.04	−0.23	0.00	−0.17	0.04	−0.24	0.00	−0.19	0.02	−0.26	0.00
hsa-miR-29B	−0.55	0.00	−0.47	0.00	−0.25	0.01	−0.28	0.00	−0.24	0.00	−0.22	0.00
hsa-miR-548d-3p	−0.19	0.04	−0.14	0.05	−0.53	0.00	−0.39	0.01	−0.49	0.00	−0.50	0.00
hsa-miR-604	−0.25	0.03	−0.37	0.00	−0.53	0.00	−0.37	0.00	−0.48	0.00	−0.30	0.01
hsa-miR-335	−0.47	0.00	−0.51	0.00	NA	NA	NA	NA	−0.17	0.02	−0.19	0.01
hsa-miR-379	−0.32	0.00	−0.35	0.00	NA	NA	NA	NA	−0.39	0.03	−0.46	0.01
hsa-miR-27b	−0.34	0.00	−0.49	0.00	NA	NA	NA	NA	−0.30	0.00	−0.15	0.04

Further, we also analyzed miRNAs dysregulated in CP and PDAC specific to tissue or blood. In tissue, 43 (22 downregulated and 21 upregulated) and 66 miRNAs (35 downregulated and 31 upregulated) are significantly modulated in CP and PDAC respectively and 40 (20 upregulated and 20 downregulated) were commonly dysregulated in both CP and PDAC (Supplementary Table S1). Interestingly in blood, 69 miRNAs (29 downregulated and 40 upregulated) were commonly altered in CP and PDAC as compared to normal samples (Supplementary Table S2).

### Deciphering progressively dysregulated genes across CP to PDAC

**Figure 2:**
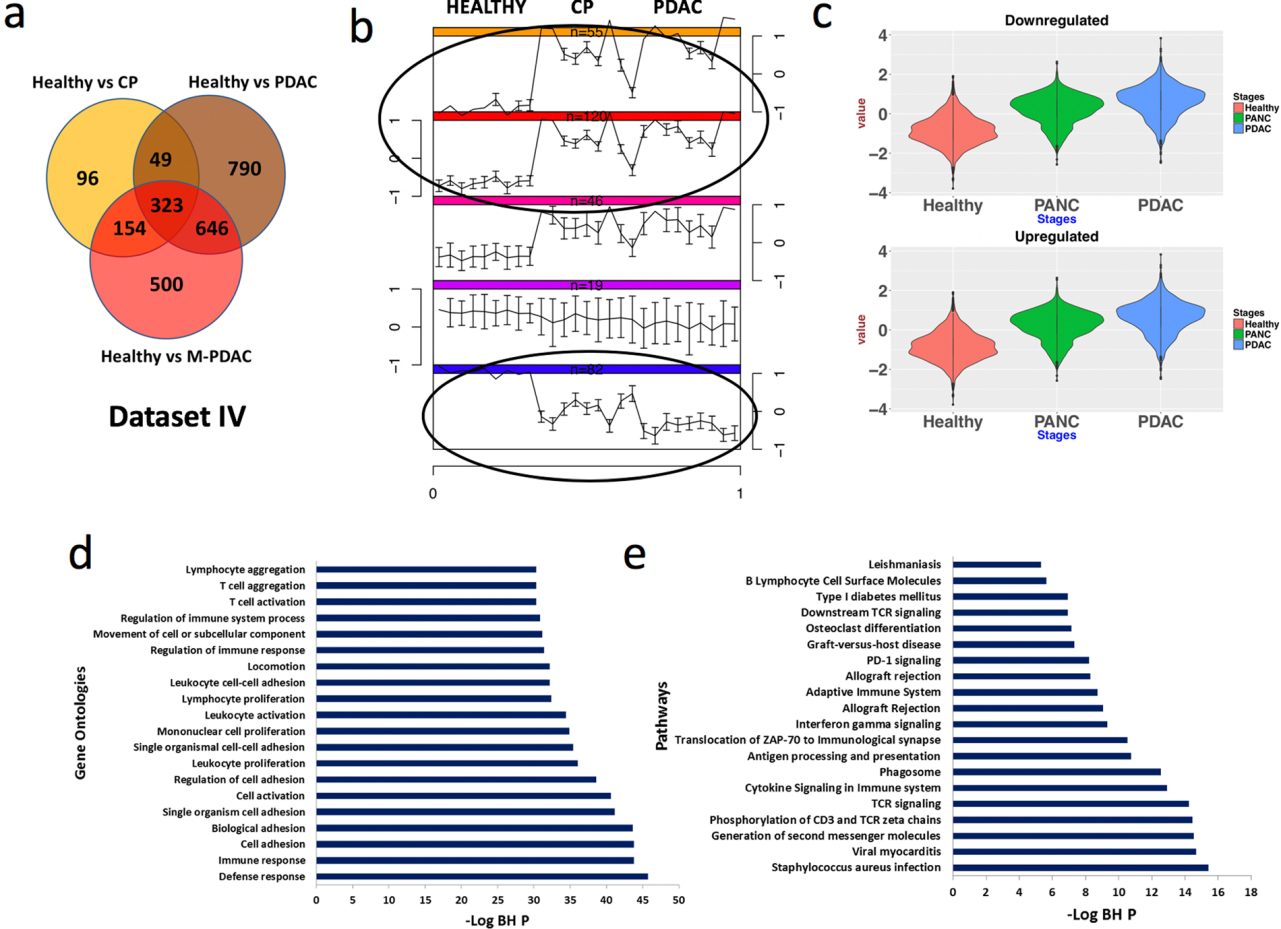
Identification of dysregulated genes in tissue datasets. (**a**) Venn diagram of the common mRNAs in HC vs CP; HC vs PDAC and HC vs MPDAC comparisons. (**b**) Partitioning of samples on the basis of expression profile of genes using SOM clustering method. We identified two strikingly opposite expression patterns (black ellipses). ‘n’ represents the number of samples clustered. (**c**) Violin plots representing the two patterns observed in SOM partitioning. (**d**) Top 20 GO categories of DE mRNA common across all conditions (blue bar). (**e**) Top 20 Pathway categories of DE mRNA common in DE progressive mRNA in all conditions.

Supervised and unsupervised analyses were performed on normalized gene expression data. The gene expression profiles were obtained from nine healthy individuals, nine patients with PDAC, nine patients with CP and nine patients with MPDAC. These samples were collected after surgical resection and represented as CP, PDAC and MPDAC respectively in this study. PCA results showed good separation of HC, CP, PDAC and MPDAC samples along the first three PCs (Supplementary Fig. S2). Interestingly, 11.43% variance was found to be contributed by PC1. In comparison to HC, 622 DE genes were identified in CP, 1808 DE genes were identified in PDAC and 1623 DE genes were identified in MPDAC. As seen in Venn diagram, 372 genes were common across CP and PDAC groups; 477 genes across CP and MPDAC groups while 969 genes common across MPDAC and PDAC groups (Fig. 2a). Notably, 323 genes (234 upregulated and 89 downregulated) were commonly dysregulated in all the groups i.e. CP, PDAC and MPDAC. Further, we generated a visualization of the data by clustering subjects based on gene expression patterns using self-organizing maps (SOM) analysis (Fig. 2b). This analysis allows for the recognition of patterns in the gene expression data as well as those which are instrumental in the classification of samples. The SOM analysis output showed a striking difference in gene expression patterns amongst patient clusters illustrating the upregulation and downregulation of genes in each group. This corroborates with the notion that patients have differential gene expression with concomitant disease progression. Each patient gene expression cluster is represented by the average expression and the variation in the gene expression pattern can be visualized by the error bar. 175 upregulated and 82 downregulated genes were further filtered using log fold change (logFC) >1.5 criteria to identify genes depicting progressive expression correlation with disease status. We define a progressive upregulation pattern of genes as a gradual increase in expression from the HC to CP, and then from CP to PDAC. In other words, the gene expression values were the lowest in HC, higher in CP, and the highest in PDAC. A similar trend was observed for the downregulated genes, as the expression for the downregulated genes decreased from HC to CP, and further to PDAC. 217 DE genes (152 upregulated and 65 downregulated) were found to have progressive change from HC to CP to PDAC. The expression pattern is depicted by violin plots where the median expression decreases from HC to PDAC in the progressively downregulated gene signature, whereas the median gene expression increases from HC to PDAC groups in the progressively upregulated gene signature established for this study (Fig. 2c and heatmap in Supplementary Fig. S3). These progressively upregulated DE genes depicted significant enrichment in gene ontology (GO) categories linked to defense and immune responses, cell and adhesion mechanisms and leukocyte/lymphocyte proliferation (Fig. 2d).

Further pathway analysis of the genes that are downregulated in the CP and PDAC groups were involved in glutamine degradation, *Staphylococcus* *aureus* infection, viral myocarditis, TCR signaling, phagocytic activity, generation of second messenger molecules, cytokine signaling in immune system, phosphorylation of CD3 and TCR zeta chains, adaptive immune system and antigen processing and presentation (Fig. 2e). The analysis reveals that the common dysregulated pathways in CP and PDAC groups are associated with the dysregulation of multiple immune response-related pathways indicating a key role of the immune system in onset of PDAC or progression of CP to PDAC.

### Exploring the gene and miRNA axis associated with disease progression

**Figure 3:**
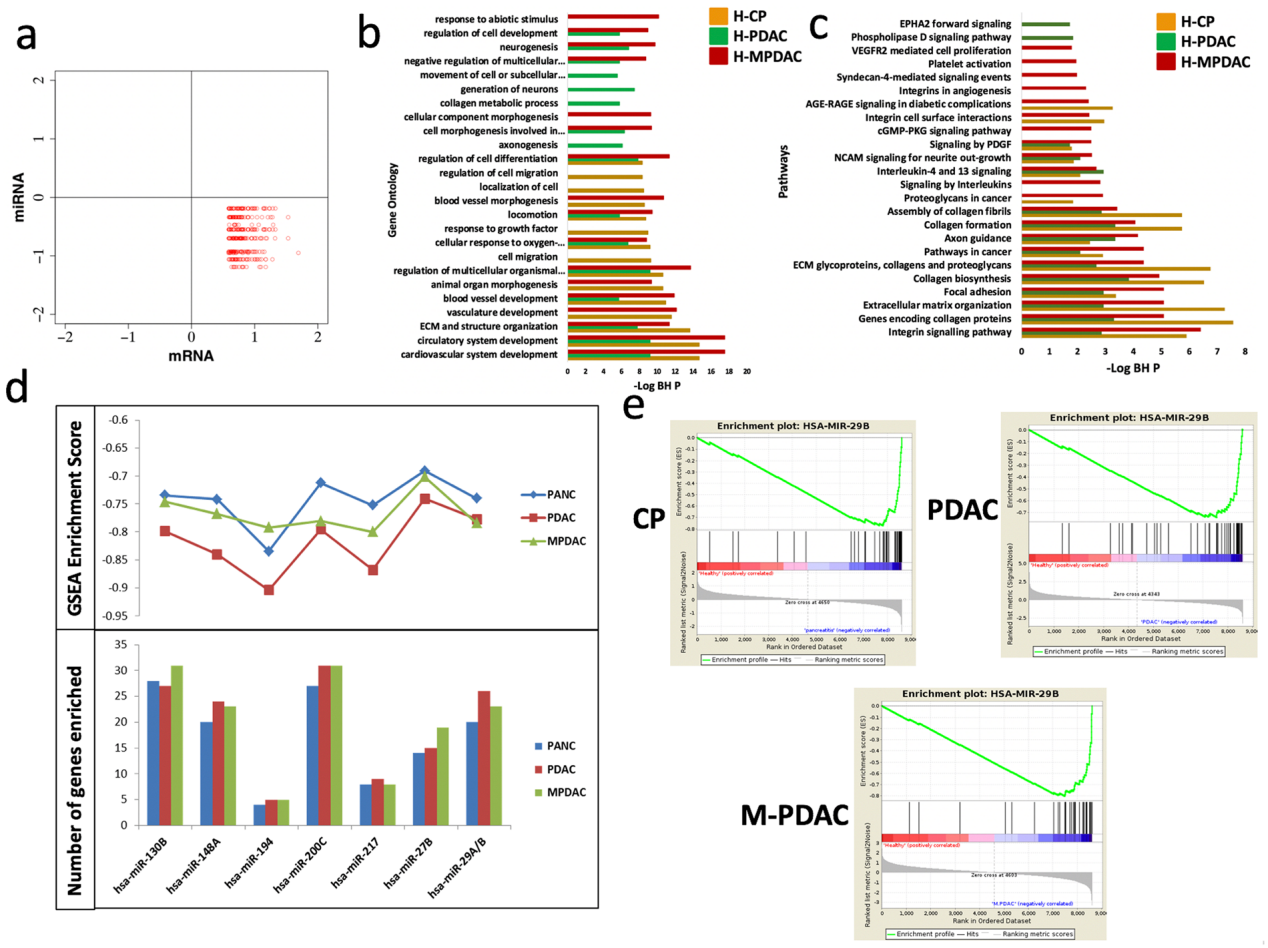
Study of miRNA-mRNA interactions in datasets and their enrichment. (**a**) Biplot showing inverse relationship between miRNA and mRNA. Along x-axis are the Log-fold change of genes and Log-fold change of interacting miRNAs are along y-axis. Upregulated genes are denoted in red. (**b**) Top 30 GO categories of negatively regulated genes by miRNA in HC-CP condition (orange bar) vs HC-PDAC condition (green bar) vs HC-MPDAC condition (red bar). (**c**) Top 20 Pathway categories of significantly affected by genes targeted by miRNA dysregulated in HC-CP condition (orange bar) vs HC-PDAC condition (green bar) vs HC-MPDAC condition (red bar). (Note: The top categories were arranged in descending order as obtained from the negatively regulated mRNAs from HC vs PDAC comparison). (**d**) Enrichment score for miRNA (P value < 0.05) shown in line graph and number of genes enriched in each condition shown in bar graph (**e**) GSEA enrichment of negatively regulated genes in HC-PDAC condition by miRNA-29a.

The meta-analysis of transcriptome and regulatory miRNA expression resulted in a large list of DE molecules associated with CP and PDAC. Even though the results provide a significant starting point for understanding disease pathophysiology, it is difficult to identify disease-driving molecules from a long list of genes alone. Considering this, the interactions between microRNA and genes may be instrumental in understanding various regulatory mechanisms. The crosstalk between expressed genes and miRNAs was retrieved from experimentally derived miRNA-gene pairs in the MSigDB and MIRTarBase databases. To understand this complexity and identify the gene-miRNA interactions in PDAC, we analyzed the gene and miRNA expression profiles of CP and PDAC patients. 370 genes were identified to interact with 13 miRNAs from the PDAC meta-gene signatures. Hence, expression of these genes is potentially regulated by miRNAs. The inverse proportionality postulate between miRNA and gene expression was tested by generating biplots of genes with miRNA expression. Bimodal gene-miRNA interaction in CP and PDAC samples depicted that 132 genes were upregulated by downregulation of their 10 upstream regulatory miRNAs (Fig. 3a). 132 genes that are counter-regulated by 10 miRNAs in progression of PDAC depicted significant over-representation to biological processes such as cardiovascular and circulatory system development, extracellular matrix organization (ECM) and extracellular structural organization (Fig. 3b). Specific biological processes namely cell migration and localization were specific to CP whereas axonogenesis and collagen metabolic processes are significantly expressed in PDAC stages. We found that regulation of cell development is upregulated in malignant and non-malignant stages whereas morphogenesis of blood vessels and cellular components and vasculature development are specific to malignant stages. The upregulated pathways targeted by the common downregulated miRNAs are linked to focal adhesion, ECM receptor interaction, ECM organization and collagen formation (Fig. 3c). EPHA2 forward signaling and Phospholipase D signaling pathway are specifically upregulated in PDAC whereas VEGFR2 mediated cell proliferation, cGMP-PKG signaling pathway, platelet activation and integrins in angiogenesis are upregulated in MPDAC stage.

Transcription factors (TFs) are commonly deregulated in the pathogenesis of human cancer and they can be used as drug targets^14^. To better understand the pathogenesis of PDAC, we further performed TF enrichment analysis. Interestingly, the analysis identified enrichment of multiple TFs including TCF4, WT1, CEBPD, CEBPB, SUZ12, and ZFP281 across all stages of pancreatic cancer progression (Supplementary Fig. S4a). CEBPB and CEBPD are CCAAT/Enhancer binding protein have biological roles in inflammation^15^. WT1 is an oncogene and is important for normal cellular development and cell survival^16^. SUZ12 has been known to promote the proliferation of gastric cancer and also in metastasis^17^. The analysis also identified enrichment of eight TFs including MYB, NUCKS1 NRF2 and SOX2 targets in PDAC and MPDAC stages (Supplementary Fig. S4b). NRF2 and SOX2 TFs are key for tumor progression and inhibiting them effectively reduces metastasis^18–21^. Additionally, over-representation of multiple TF targets were found for only PDAC (e.g. KDM2B, HNF4A) and MPDAC (PU1, TP63) stages (Supplementary Fig. S4c). KDM2B^22,23^ and HNF4A^24^ are potential therapeutic targets and well-known for their role in cancer progression. The upregulated TFs in MPDAC are known to be involved in shorter survival of cancer patients (PU1^25^), metastasis (TP63^26^), vasculogenesis (FLI-1^27^) and cellular functions including cell proliferation, DNA damage and repair, apoptosis and stress response (FOXO1^28,29^).

### Enrichment of negatively regulated genes under miRNA regulatory controls

The significance of the negatively regulated genes and miRNA obtained from the meta-analysis was assessed using a gene-set enrichment analysis (GSEA) approach. The enrichment of upregulated DE genes controlled by 10 of the 13 downregulated miRNAs was assessed using GSEA (Supplementary Fig. S5). The DE genes from the three comparisons of Dataset IV viz. HC vs CP, HC vs PDAC and HC vs MPDAC, were retrieved separately for these 10 miRNAs.

The target genes for most (7 out of 10) of miRNAs involved in progression of the pancreatic cancer depicted significant enrichment in PDAC (nominal P-value ≤ 0.05) (Supplementary Table S3). Figure 3d shows the GSEA enrichment scores and the number of genes enriched to the corresponding miRNA, as depicted on the y-axis respectively. The miRNA-130b, 148a, 27b and 29a/b had a negative enrichment score >2 but the maximum increase in the number of enriched genes from CP to PDAC (1.3 fold) was observed for miRNA-29a/b (Fig. 3e).

**Figure 4:**
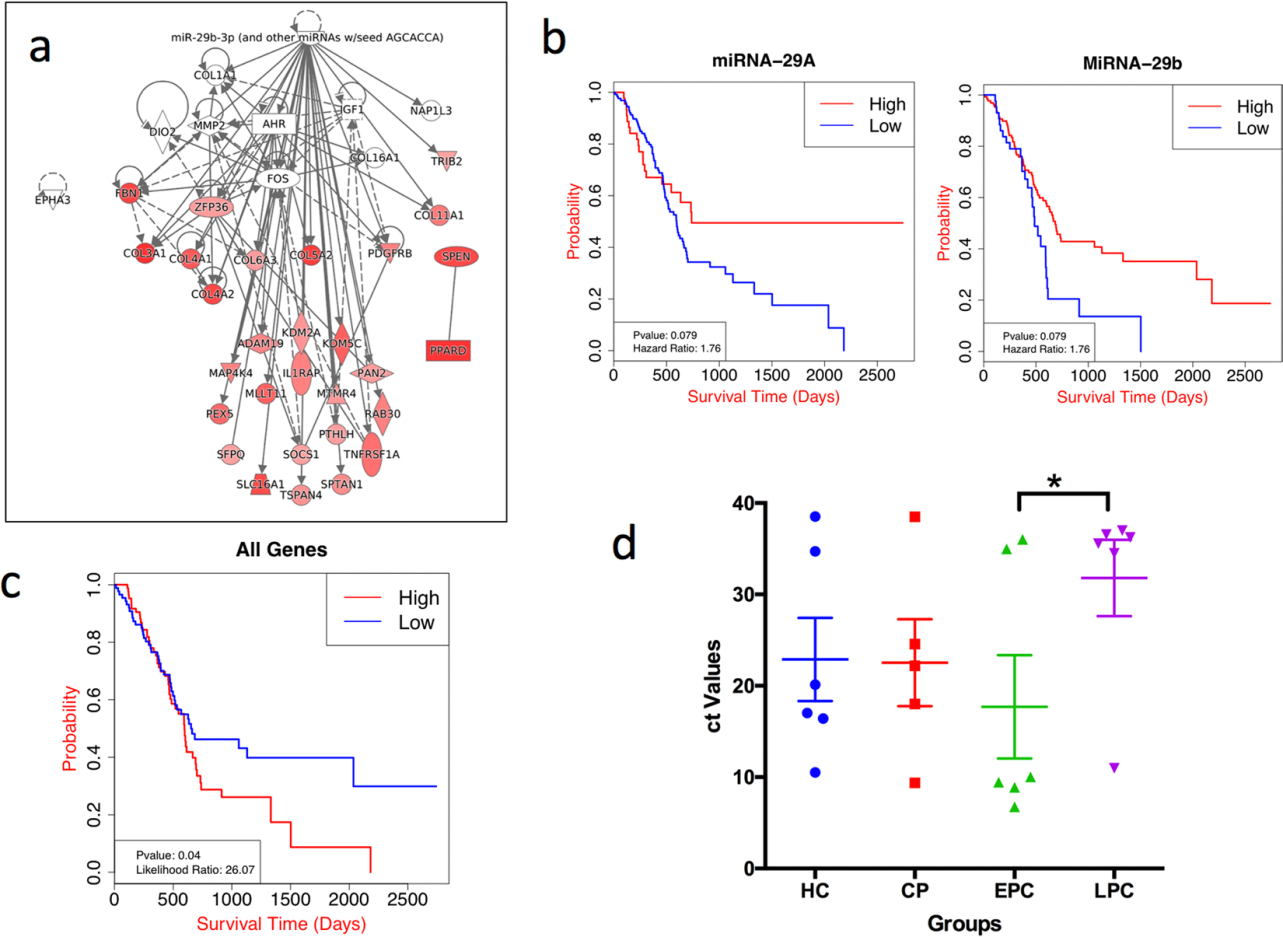
Regulatory network, survival analysis and validation of miRNA-29a/b. (**a**) Co-expression network representing progressive increase in the negatively regulated genes by miRNA-29a/b miRNA in HC vs PDAC as compared to CP. The networks were generated through the use of IPA (QIAGEN Inc., https://www.qiagenbio-informatics.com/products/ingenuity-pathway-analysis ^30^. (**b**) Survival curve of miRNA-29a and miRNA-29b with TCGA cohort divided at 75^th^ percentile. (**c**) Survival curve of additive effect of 15 mRNA markers, identified from independent survival analysis, with TCGA cohort divided at median. (**d**) Ct values obtained by RT-PCR analysis for miRNA-29a in the serum sample of CP (chronic Pancreatitis), EPC (Early Pancreatic Cancer) and LPC (Late Pancreatic cancer) as compared to HC. The mean and standard error of mean of Ct values from each group is shown along with individual samples denoted by a dot. The significant differences (p value < 0.05) between groups are marked with asterisk.

To represent the influence of miRNA-29a/b in the advancement of disease, we next visualized the effect of the miRNA on the regulation of genes associated with the progression of pancreatic cancer. The set of 132 upregulated genes that are targets of 10 miRNAs were used for this purpose, similar to GSEA enrichment analysis. We selected the mRNA targets of miRNA-29a/b for the CP group and used ingenuity pathway analysis (IPA) (QIAGEN Inc., https://www.qiagenbio-informatics.com/products/ingenuity-pathway-analysis)^30^, to build the knowledge-based network using miRNA-29a/b as a seed (Fig. 4a). Further, we added the mRNAs that were targeted by miRNA-29a/b in PDAC, to the already built interaction network from CP and miRNA-29a/b. From the GSEA enrichment we already knew that there were more genes that were regulated by miRNA-29a/b with the progression of disease and the knowledgebase network obtained from IPA could validate the network enrichment with the disease progression from CP to PDAC. Few DE genes (11 upregulated genes) in CP and PDAC groups were common targets of the miRNA-29a/b.

### Survival analysis with the identified key regulators in progression of pancreatic cancer

The significance of the miRNA-29a/b in the progression of the pancreatic cancer was further tested using survival analysis in the the cancer genome atlas (TCGA) database. The samples were partitioned into two groups at 75^th^ percentile for the selected miRNA and survival analysis was performed on the two clusters (Fig. 4b). The results showed that both miRNA-29a and miRNA-29b were able to clearly discriminate between better versus poor survivors (P-value 0.06 and 0.01, respectively), indicating their prognostic role in PDAC. Low miRNA-29a, 29b expression is associated with shortened survival time. Also, the hazard rate of the samples with downregulated miRNA-29a and 29b was as high as 1.76 and 2.29 respectively, as calculated by cox proportional hazard model. Further, we found that 15 of the 53 mRNAs targeted by miRNA-29b have hazard ratio of >2 at significant P value < 0.05 (Supplementary Fig. S6). Overexpression of MAP4K4 and AKAP13 are associated with significant shortened survival time in PDAC patients. MAP4K4 a serine/threonine kinase plays significant role in immunity, inflammation, metabolic and cardiovascular diseases and have been recognized as actionable cancer therapeutic target^31,32^. Genetic variation and expression of AKAP13, a protein kinase, is associated to regulation of cancer and response to cardiac hypertrophy^33,34^. From other genes CHSY1^35^, TRIB2^36^, IL1RAP^37^, and KDM2A^38^ were also known to be actionable cancer therapeutic targets. The combined survival analysis of all the 15 genes has a likelihood ratio of 26 and Hazard ratio of 2 at a significant P value of 0.04 (Fig. 4c).

### Validation of the meta-analysis derived miRNA signature

From the meta-analysis, network analysis, and survival analysis data the most potent miRNA that appeared promising for further validation was miRNA-29a and miRNA-29b. q-RT PCR analysis was employed on sera samples from a panel of 23 patients (6 HC, 5 CP, 6 Early PDAC [EPC] and 6 Late PDAC [LPC]). C_t_ values are inverse to the amount of miRNA detected. The Ct counts for miRNA-29a was higher in the LPC cases than the HC and CP cases, indicating that the PDAC subjects showed lower abundance of miRNA-29a specifically towards late stage of the disease (Fig. 4d). The figure represents the Ct values for the miRNA-29a amount in different stages of cancer with mean and standard error of mean in each group. We could not find any significant differences in comparing the Ct values in between HC versus CP (P value: 0.411), HC vs EPC (P value: 0.23) and HC vs LPC (P value: 0.29) stages because of very small amount of miRNA detected in samples. However, the Ct values were significantly different between EPC vs LPC (P value: 0.04).

## Discussions

The identification of a molecular signature for the pancreatic cancer progression in humans is quite an intriguing yet challenging field. However major problems persist, such as the lack of patient samples and insufficient studies comparing early stages of cancer to matched healthy cases. As obtaining tissue biopsies will continue to be a primary limitation in studies of solid tumors located in an inaccessible organ, tissue-based markers are not a practical choice for following disease progression longitudinally. Elucidating the markers that will reflect the progression of disease and specifically identify the cases with an underlying disease without any clinically evident symptoms, is quite challenging. Thus, our study aimed to obtain the circulating markers that could identify the progression of pancreatic cancer and can in turn, effectively detect any incipient disease in high risk individuals. For the purpose to identify the major regulators of disease from CP and early stages of PDAC, we selected only those studies that have microarray data from CP and multiple stages of PDAC along with HC. Further, we obtained the miRNA and mRNA studies from blood as well as tissue in order to obtain the circulating markers that are consistent with the alterations in tumor expression and have associations with the progression of PDAC in humans. As the source of miRNA in blood could be from multiple tissues and organs, hence incorporating pancreatic cancer tissue studies in our meta-analysis converged the list of markers specifically altered and secreted from pancreas during PDAC development.

These analyses specifically target the identification of key miRNAs involved in the progression of this disease. For this purpose, the miRNA studies with the samples from HC, CP and PDAC patients procured from the various groups were obtained. The preprocessing of the microarray data using Z normalization brought the data from many studies into consistent ranges that allowed the identification of persistently dysregulated miRNAs in CP and PDAC in both tissue and blood samples. The HC vs PDAC comparison has a large set of miRNAs common in both tissue and blood, but CP subjects overlapping the HC and PDAC subjects (Supplementary Fig. S2) have only a few candidate DE miRNAs. The overlap between CP and PDAC was only 13 miRNAs, all being consistently expressed in tissue as well as blood. Also, a mRNA study of patients from HC, CP, PDAC and MPDAC groups was included. The mRNA study was incorporated to facilitate the understanding of the miRNA-mRNA interaction axis and its role in the progression of the disease. The GO analysis of the DE mRNAs shows that the progressive DE genes dysregulate the immune system of the host with concomitant progression of disease. It has been shown previously that spontaneous cancer development is facilitated by the imbalance of the local immune microenvironment by the tumor cells in the pancreas^39^. The DE genes shows significant downregulation in the glutamine degradation pathways, the amino acid (AA) to which most cancer cell have an addiction^40,41^. PDAC cells have also been reported to have an AA shortage and the metabolic reprogramming to provide the branched-chain AAs starts in very early stages of the PDAC^42,43^. cGMP-PKG signaling pathways expression, related to malignant stage of PDAC, have been known to determine the stimulatory and inhibitory actions in cancer cells and play an important role in tumorigenesis^44^. We also report the unique and common TFS in/across malignant versus non-malignant PDAC stages. The expression of TFs unique to stages can be used as markers to identify the progression of diseases whereas the markers common between two stages can be used as therapeutic targets e.g. NRF2 and SOX2 for metastasis and SUZ12 for progression of PDAC disease.

We performed a multi-step approach integrating the cross-talk between transcriptome and regulatory miRNA as well as an interactive pathway network analysis to identify the key regulatory miRNA, circulating in the blood, associated with the progression of PDAC. This analysis is important for understanding the controlling cascades and cross-talk between gene expression and regulatory mechanisms. The miRNAs identified in this analysis have been shown previously to be dysregulated in cancer. For example, miRNA-130b has been previously identified as a prognostic marker, which on over-expression, inhibits cell proliferation and invasion in PDAC by targeting STAT3^45,46^. The downregulated expression of miRNA-148a has been detected in various cancers including gastric, colorectal, pancreatic, liver, esophageal, breast, non-small cell lung and urogenital system cancers^47^. miRNA-217 functions as a potential tumor suppressor^48^ and also, the overexpression of miRNA-200c plays an inhibitory role in human PDAC stem cells^49^. miRNA-29b has been shown to critically affect cancer progression by functioning as a tumor suppressor^50^. miRNA-29a has been mentioned as a potential therapeutic agent to target PDAC as the restored expression of this miRNA blocks autophagic flux by inhibiting expression of key autophagic proteins^51^. The analysis only could identify the miRNAs that are commonly downregulated in both CP and PDAC and that might reduce diagnostic efficacy of markers. We believe with the availability of highly sensitive miRNA and mRNA quantification technologies such as droplet PCR, Nanostring, and single cell genomics in last decade provided highly sensitive tools to explore efficacy of downregulated molecules as biomarkers^52–56^.

In the past multiple studies have performed the integrative analysis on transcriptome, SNPs, the proteome and non-coding RNAs^57–59^ to identify PDAC prognostic and diagnostics markers based on molecules that are alerted across multiple genomic spaces. The identification of robust biomarkers achieved minimal success due to limited number of studies, lack of paired samples, and minimal correlation between transcriptome and proteome data. Therefore, in this study we attempted to identify robust miRNAs associated with PDAC pathophysiology and progression based on meta-analysis of miRNA, and genes as well as using their robust regulatory interaction information. Our study doesn’t incorporate additional mutation and proteome analysis due to limited availability. This information could be used to further refine the results to identify robust biomarkers of PDAC pathophysiology. Though, we believe that with our sound computational approach, we have identified some key molecules and pathways involved in pathophysiology and the progression of pancreatic cancer.

In conventional analysis, most of these miRNAs would be weighted equally in their role in progression of PDAC, whereas our systems-level analysis revealed that miRNA-29a/b plays a significantly more prominent role. The progressively decreasing enrichment score of miRNA-29a/b alongside the concomitant increasing trend in enrichment of its mRNA targets across CP to PDAC to MPDAC cases, rationalized our focus on this particular miRNA for the rest of our studies. Study of the pathophysiological role of miRNA-29 revealed that miRNA-29a is the most abundantly expressed miRNA-29 family member in the human pancreas and pancreatic stellate cells^11^. Thus, our wet-lab validation focused on identifying the expression pattern of miRNA-29a in human serum samples, which depicted a similar decreasing trend in the abundance of miRNA-29a from HC and CP to PDAC cases. From our system-biology approach and wet-lab validation, miRNA-29a emerged as a potential circulating marker for the progression of PDAC. Also, we could identify MAP4K4 and AKAP13 as the prognostic markers from the mRNAs targeted by miRNA29a. These markers could be further tested for their efficacy for their use as prognostic markers or as therapeutic targets in pancreatic cancer.

GSEA and network analysis revealed that the interacting mRNAs for the miRNA-29a/b were involved in regulating the ECM matrix organization and focal adhesion of cells, thereby indicating a potential role of miRNA-29a/b in regulating the pathways essential for progression of PDAC. miRNA-29 also plays a critical role in regulating tumor stromal deposition and cancer growth, thereby implying that modulation of miRNA-29 expression may be a therapeutically beneficial target to enhance the efficacy of chemotherapy for the improved treatment of PDAC^11^.

## Conclusions

In the present study, our goal was to identify the circulating miRNAs which play a crucial regulatory role in driving the progression of PDAC. Using multi-omics studies, we were able to pin down one such miRNA family, miRNA-29, and more specifically, its family member miRNA-29a, as a potential prognostic candidate marker for PDAC progression. Considering the associated trends in miRNA-mRNA interactions across various disease statuses, miRNA-29a/b was found to downregulate the pathways associated with focal adhesion and ECM organization in the cells, which are necessary for cancer progression and metastasis. Although, both miRNA-29a and miRNA-29b were able to differentiate poor survivors from better survivors in silico, the RT-PCR results revealed better performance of miRNA-29a from serum samples in identifying PDAC from CP and discriminating early versus late PDAC. This is likely because of its higher abundance in the pancreas as compared to the other members of its family. This validation strengthened our preliminary finding that miRNA-29a has prognostic potential and can be used as a diagnostic marker for PDAC progression, however this needs to be further validated in a larger cohort study. In addition, considering its substantial role in regulating molecular pathways involved in metastasis, miRNA-29a can be used as a potential therapeutic target to complement current chemotherapeutic strategies.

## Methods

### Data collection and processing

Raw transcriptome data on miRNA and mRNA were obtained from public repositories, namely Gene Expression Omnibus (GEO)^60^ and ArrayExpress^61^, and were normalized using R statistical software and Bioconductor packages in a platform-specific manner. The information about datasets used in the study along with their sample details is listed in Table 1. The meta-analysis of plasma and tissue miRNA data was based on three published studies containing pancreatic adenocarcinoma (PDAC), Chronic Pancreatitis (CP) and healthy control (HC). CP samples are obtained from patients only suffering with CP and not from adjacent tissues of patients suffering with PDAC. As the focus of the current study was to identify miRNA and genes associated with progression of CP to PDAC, we have only included studies with samples from CP, PDAC and HC. The normalized intensities were obtained from Febit microarray data from the GEO database using GEO-R approach and were Z-normalized using ClusterSim package in R^62^. The gene signatures were identified from Dataset IV with samples from HC, CP, PDAC and Metastatic-PDAC (MPDAC). The normalized intensities were obtained for Affymetrix HG_U95Av2 micrarray data from ArrayExpress and were z-normalized. Z-normalization was preferred approach for meta-analysis as it results in relatively transforming data to same scale independent of approach and platform used for generating data.

### Quality control and unsupervised analysis

The quality of the normalized array data was assessed using the arrayQualityMetrics package^63^ from Bioconductor. Technical Outliers were identified on inter-array expression distances (mean absolute distance of the M-value for each pair of arrays). Samples with greater than 10% outliers were not included in the study. Unsupervised analysis was performed using principal component analysis (PCA) to further ascertain the quality of datasets and identify outliers without biological relevance. PCA projects multivariate data objects onto a lower dimensional space while retaining as much of the original variance as possible^64^. PCA methodology captures the inherent gene expression patterns in the data and identifies the correlation among biologically distinct samples.

### Supervised analysis to identify differentially expressed molecules

Differentially expressed (DE) molecules (genes and miRNA) between **1**) CP and HC; **2**) PDAC and HC were identified using the limma package from the Bioconductor project^65^. Similarly, we have also identified differentially expressed genes associated with metastatic pancreatic cancer by comparing HC and MPDAC using similar approach. The sample groups were compared by fitting a linear model for each variable (normalized expression values) and applying empirical Bayes smoothing to identify differentially expressed molecules^66^. The miRNA with *P*- value < 0.05^51^, were considered significantly differentially expressed whereas the mRNA with absolute fold change ≥1.5 and multiple test corrected *P*-value < 0.05^51^, were considered significantly differentially expressed. The lists of differentially expressed gene/miRNA from different datasets and group comparisons (e.g. CP vs PDAC, CP vs HC, HC vs MPDAC) were compared using Venn diagrams in R.

### miRNA-mRNA counter regulation analysis

For identification of miRNA and their target that are dysregulated in PDAC, we performed miRNA target prediction analysis. The information about experimentally validated miRNA–gene interacting pairs were retrieved from the MSigDB^67^ and miRTarBase^68^ databases. miRNA play important gene-regulatory roles in multiple cancers by pairing to protein-coding genes to direct their posttranscriptional repression. Multiple genome level studies depicted counter regulation relationship between genes and miRNA expression which means that upregulation of miRNA leads to downregulation of target genes and vice versa. The miRNA-mRNA interactions were retrieved separately for the different conditions, CP and PDAC corresponding to the DE miRNA in these groups. The patterns of expression between target genes and miRNA were determined by generating biplots on the basis of fold change of differentially expressed targeted genes and interacting miRNAs.

### Pathway analysis

The analysis of the biological pathways and Ontologies for the genes was performed using ToppGene algorithm^69^. ToppGene performs functional category enrichment analysis and candidate gene prioritization based on functional annotations and a protein interactions network. ToppFun detects functional enrichment of the provided gene list based on transcriptome, proteome, regulome (TFBS and miRNA), ontologies (GO terms), phenotype (human disease and mouse phenotype), pharmacome (Drug-Gene associations), literature co-citation, and other features. The functional categories and pathways with FDR <0.05 were considered significantly enriched.

### Gene set enrichment analysis

Gene set enrichment analysis (GSEA)^70^ was performed to evaluate enrichment of dysregulated miRNAs target genes enrichment in CP, PDAC and MPDAC groups as compared to HC. The target genes expression profiles were generated from Dataset IV. The DE miRNA common between CP and PDAC groups were used for the GSEA analysis. During the enrichment analysis, the miRNA and target with opposite or counter-regulated enrichment were considered relevant as in most of cases miRNAs negatively regulate the expression of target genes. The enriched gene sets with nominal P value (NPV) <0.05 after 1000 random permutations were considered significant to eliminate the false positives hits.

### Transcription factor enrichment analysis

To explore the role of transcription factors in the pathophysiology of the pancreatic cancer we performed enrichment analysis on dysregulated genes that are targeted by differently expressed miRNAs from various comparisons: (1) HC vs. CP (2) CP vs. PDAC and (3) PDAC vs. MPDAC. We used Enrichr^71^ to perform the enrichment analysis of transcription factors. Enrichr is a comprehensive resource for pathways, Gene ontology categories and curated gene sets for enrichment analysis. Enrichr has multiple resources for transcription factor enrichment including ChEA^72^, TRANSFAC^73^, ENCODE^74^, ARCHS4^75^. In this study, we have used ChEA database for enrichment analysis that contain putative targets for transcription factors based on experimental profiling of transcription factors binding sites. The transcription factor with multiple test corrected P value < 0.01 are considered significantly affected.

### Network analysis

To further understand the molecular mechanism of PC progression, we performed a system biology analysis on constitutively altered transcripts across various pancreatic disease status (HC, CP, PDAC and MPDAC). The systems biology analysis was performed using a master regulator approach that assists in identifying the key transcriptional regulators that might be responsible for the progression of disease. Initially, we generated an interactive network of genes and miRNA using Ingenuity Pathway Analysis (IPA) (Qiagen) software (QIAGEN Inc., https://www.qiagenbioinformatics.com/products/ingenuitypathway-analysis)^30^. The software uses literature curated Protein-Protein, Protein-DNA and Protein-RNA interactions along with genes and miRNA interactions to generate interactive network. We developed separate networks for CP, PDAC and MPDAC groups of miRNA and target genes that are dysregulated in each of these statuses. To determine the relative importance of gene-miRNA interaction in disease progression from CP-PDAC-MPDAC, we performed hierarchical network analysis. The hierarchical network analysis identifies miRNA and their target genes depicted significant increase in differential expression progressively from CP to PDAC and MPDAC.

### Survival analysis

To determine the association of key miRNAs with survival in PC, we performed survival analysis using the TCGA database (https://cancergenome.nih.gov/). The survival analysis was performed on PDAC miRNA of 185 patients. Survival analysis was performed on the basis of individual miRNA expression using the Kaplan-Meier (K-M) approach^76^. The normalized expression data for each gene was divided into high (upper quartile) and low (lower quartile) groups. The survival analysis was performed using K-M analysis from survival package in R. The results of the survival analysis were visualized using K-M survival curves with log rank testing. The results were considered significant if the P value from the log rank test were below 0.05. The effects of miRNA on the event were calculated using univariate Cox proportional hazard model without any adjustments. Further, to identify the prognostic gene marker from the genes targeted by miRNA29b, we performed survival analysis of the same using TCGA database (https://cancergenome.nih.gov/). The survival analysis for genes was performed on PDAC miRNA of 179 patients. The analysis is performed using K-M approach and univariate Cox proportional hazard model as discussed above. The normalized expression data for each gene was divided into high and low groups based on median. The association with survival is considered significant i.e. P value from the log rank test below 0.05.

### Assessment of relative mi-RNA levels in patient sera

RNA was isolated from the serum of 23 subjects (6 HC, 5 CP, 6 Early PDAC [EPC] and 6 Late PDAC [LPC]) using MAGMAX RNA Purification kit. Proteinase K digestion and DNase treatment were carried on the sample before eluting the total RNA as per the manufacturer’s protocol. 100ul volume was taken for each serum sample and the concentration of total RNA ranged from 0.5–2.0 ng/ul. cDNA was prepared using TaqMan Advanced miRNA cDNA synthesis kit. TaqMan advanced miRNA assays (Thermo Fisher Scientific) were designed for hsa-miRNA-29a. Each serum sample was analyzed in independent triplicates. Ct values of miRNA-29a were plotted for each sample with mean along with standard error of mean. The significance of difference in miRNA-29a expression across groups was calculated using Wilcoxon test in ggpubr R package.

## Electronic supplementary material

Supplementary figures

Supplementary Table S1

Supplementary Table S2

Supplementary Table S3
