# Supplementary material for: Systems Biology Approach to Identify Novel Genomic Determinants for Pancreatic Cancer Pathogenesis
Source: Sci Rep. 2019 Jan 15;9:123. doi: 10.1038/s41598-018-36328-w (PMC6333820; doi:10.1038/s41598-018-36328-w)
Supplement: Supplementary file 1 — Supplementary figures [file 41598_2018_36328_MOESM1_ESM.pdf]

Systems Biology Approach to Identify Novel Genomic Determinants for Pancreatic Cancer Pathogenesis

Indu Khatri[a], Koelina Ganguly[b], Sunandini Sharma[b], Joseph Carmicheal[b], Sukhwinder Kaur[b], Surinder K. Batra[b, *], Manoj K. Bhasin[a,*]

[a] BIDMC Genomics, Proteomics, Bioinformatics and Systems Biology Center, Beth Israel Deaconess Medical Center, Boston, MA

[b] Department of Biochemistry and Molecular Biology, University of Nebraska Medical Center, Omaha, Nebraska

***Corresponding Authors**

Manoj K. Bhasin, PhD
E-mail: mbhasin@bidmc.harvard.edu

Surinder K. Batra, PhD
E-mail: sbatra@unmc.edu

Supplementary Figures

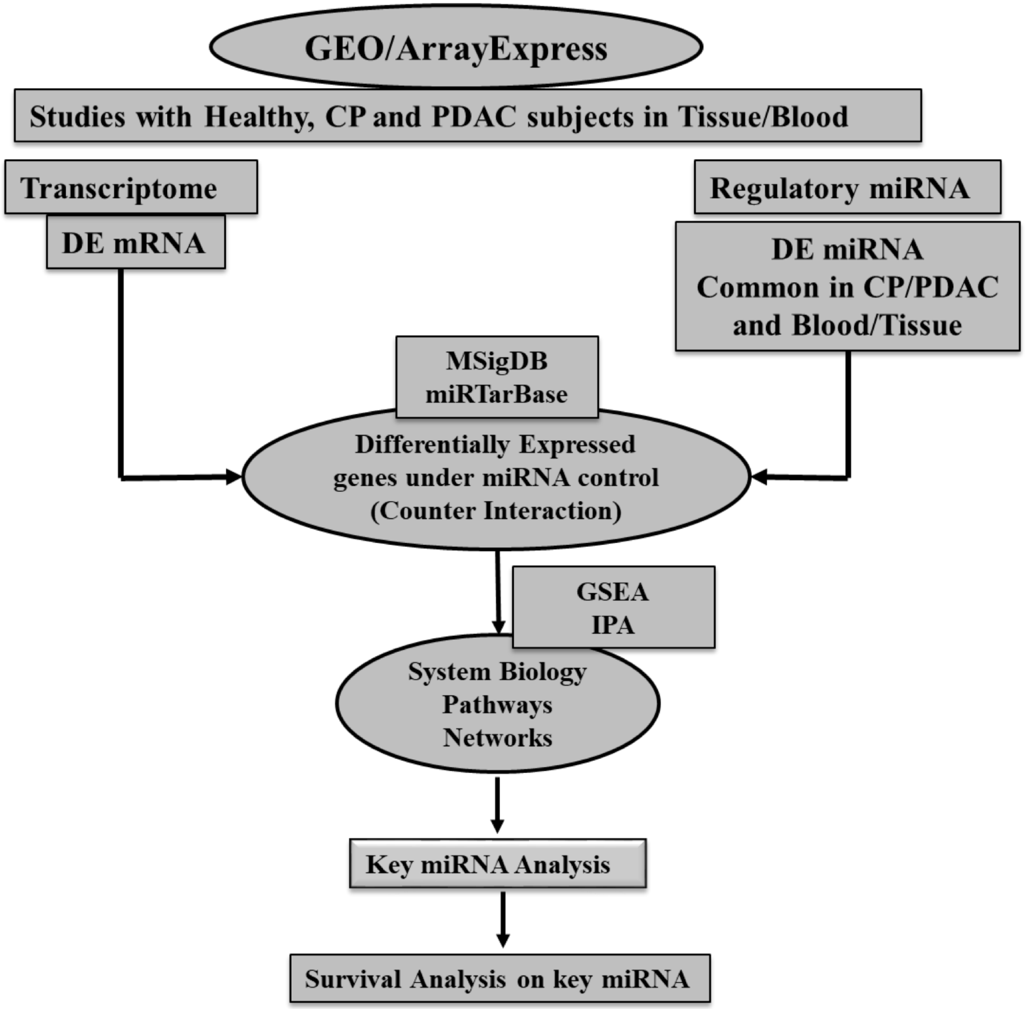

Supplementary Figure S1: Flow chart explaining the complete methodology

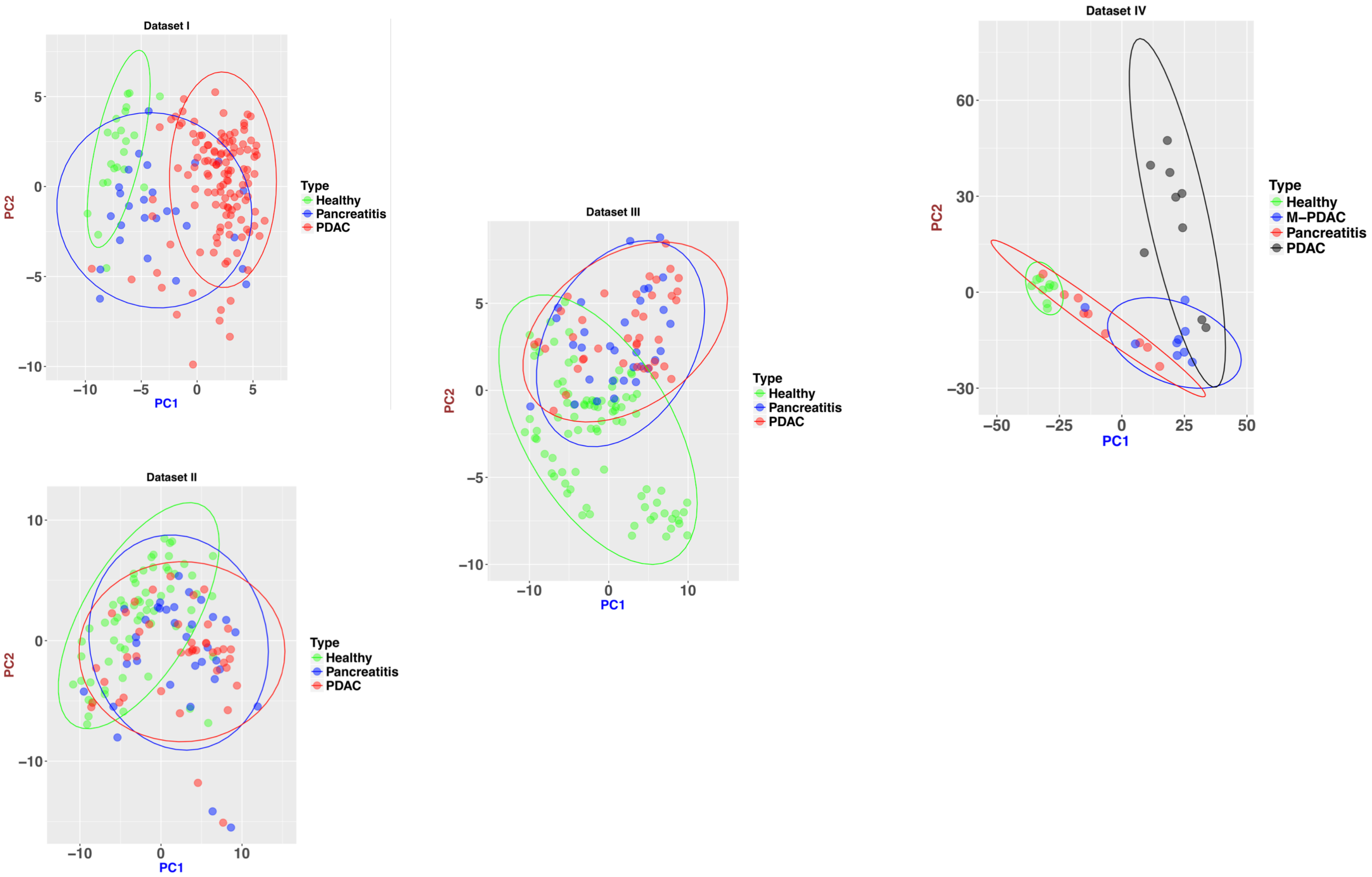

Supplementary Figure S2: PCA plots of all the four datasets used in the study.

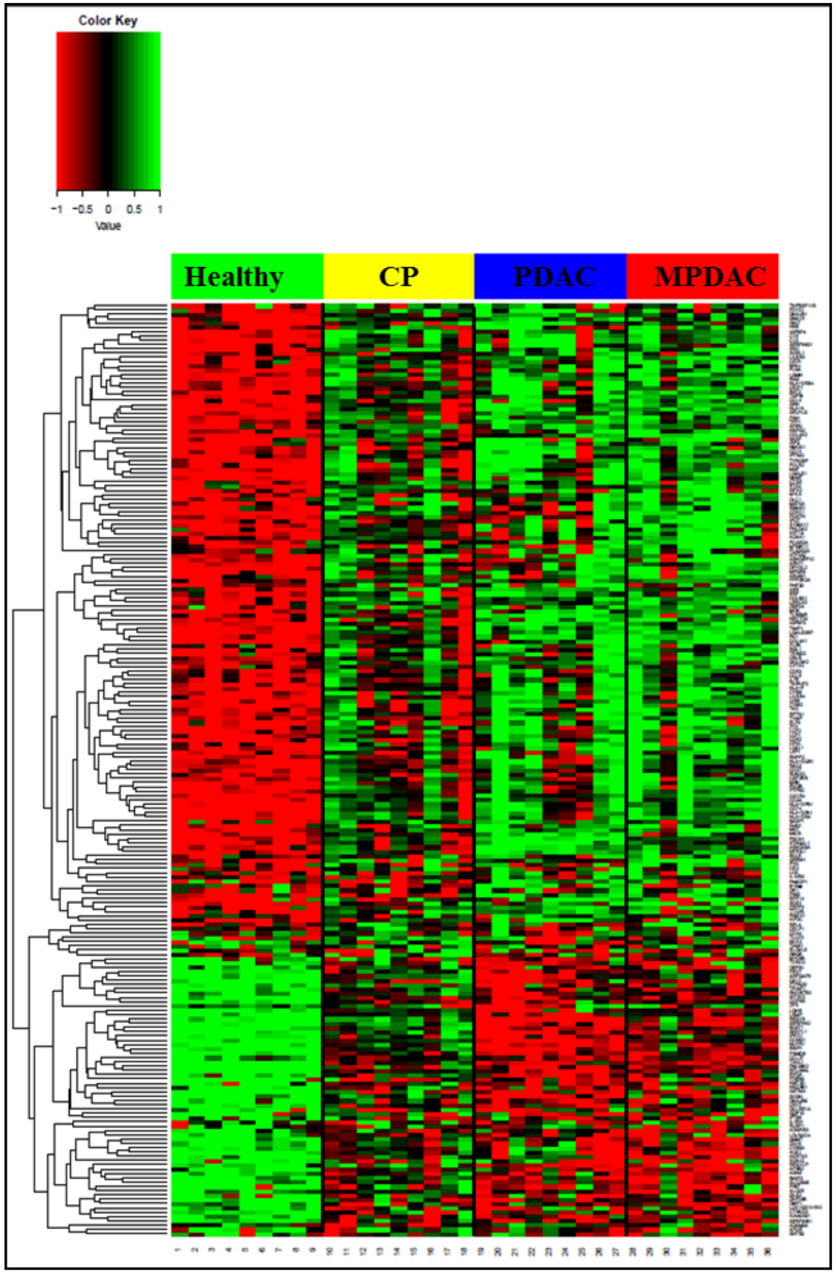

Supplementary Figure S3: Heatmap of the common mRNAs with progressive alteration in expression (upregulation/downregulation) between HC vs CP; HC vs PDAC and HC vs MPDAC comparisons.

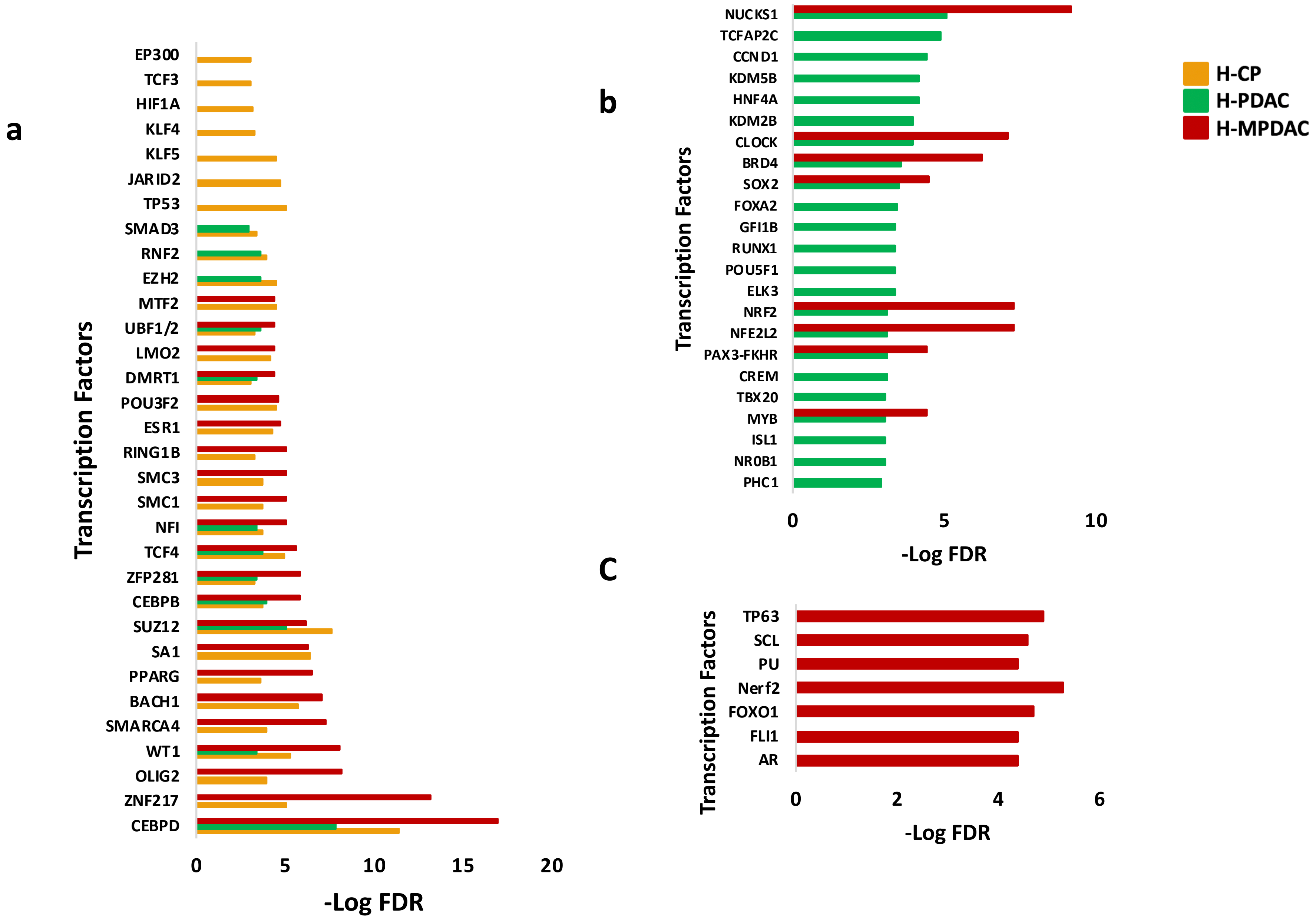

Supplementary Figure S4: Transcription Factor enrichment of regulated genes in HC-PDAC-MPDAC condition by DE miRNA common in CP and PDAC conditions.

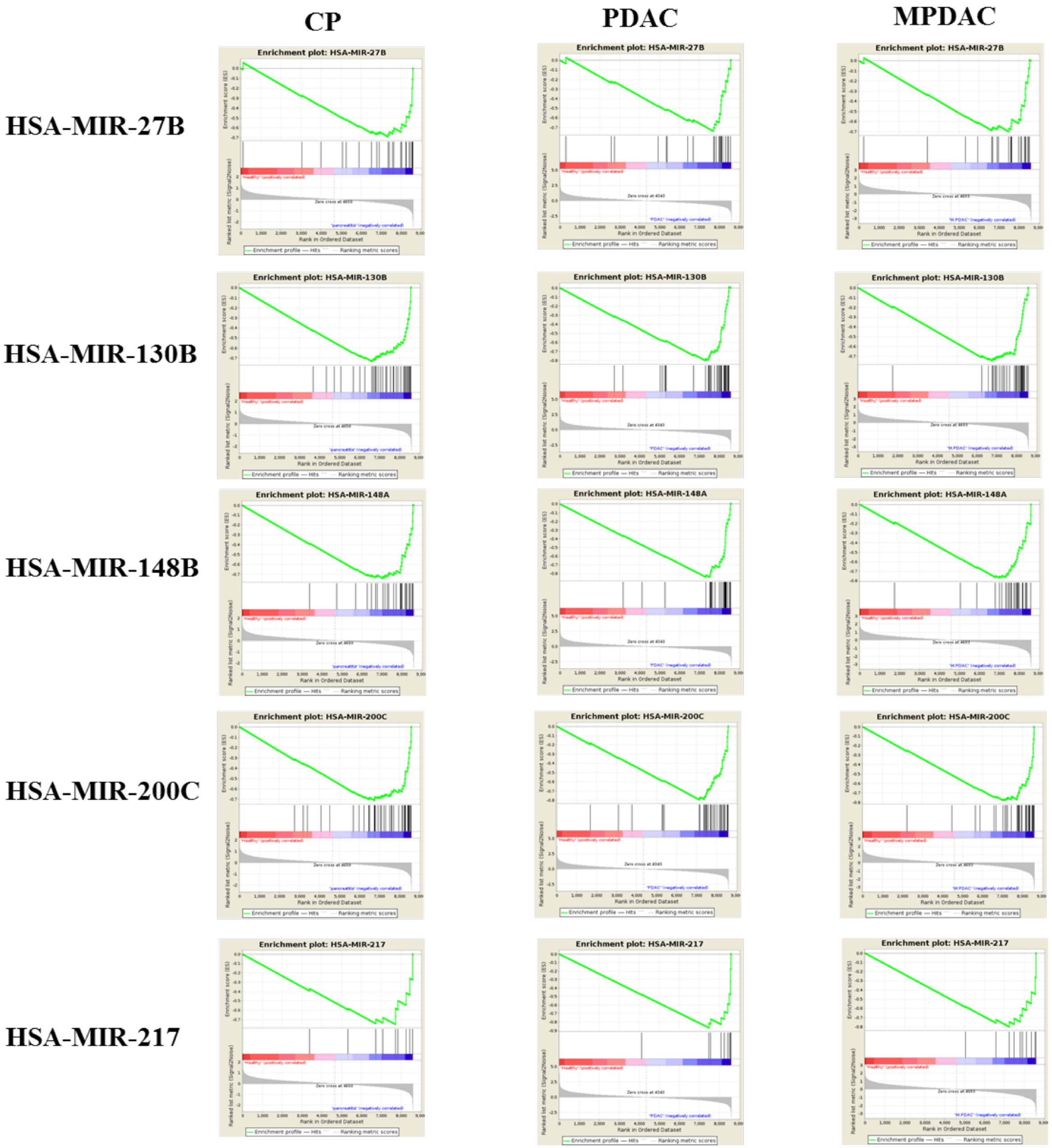

Supplementary Figure S5: GSEA enrichment of negatively regulated genes in HC-PDAC condition by DE miRNA common in CP and PDAC conditions.

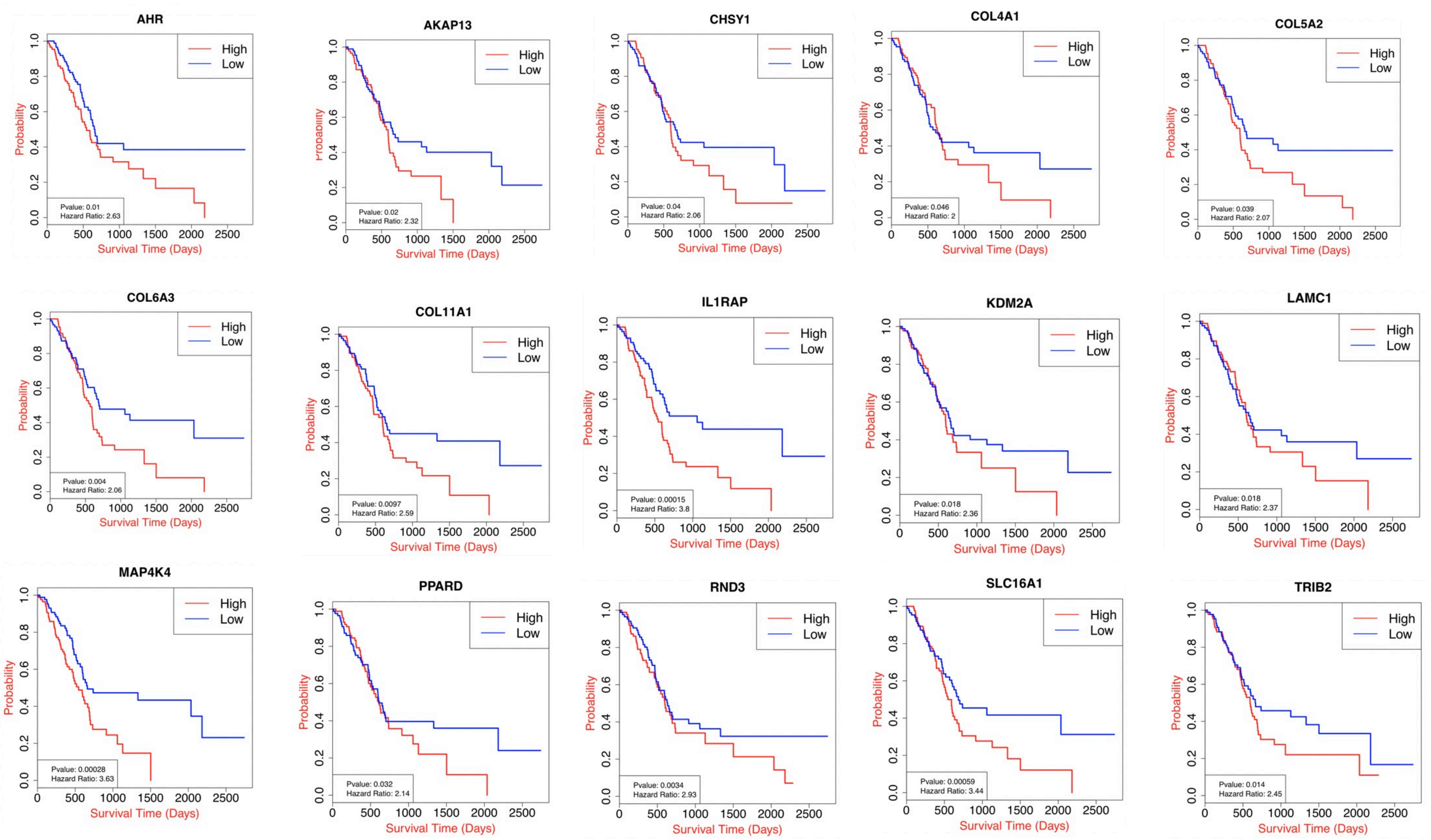

Supplementary Figure S6: Survival curves of mRNA regulated by miRNA-29b with TCGA cohort divided at median. The 15 mRNAs are significant with hazard ratio of >2 out of 35 mRNAs regulated by miRNA-29b .
